# Perceptions of Health Professionals on the Implementation of Integrative and Complementary Practices at a University Pediatric Hospital in Brazil: A Qualitative Interview Study

**DOI:** 10.1177/15347354231192004

**Published:** 2023-08-29

**Authors:** Marc Tröndle, Danton Matheus de Souza, Rita Tiziana Verardo Polastrini, Vicente Odone Filho, Georg Seifert, Wiebke Stritter, Sarah B. Blakeslee, Ana Lucia dos Santos Teco Mucci, Paula Lage Pasqualucci

**Affiliations:** 1 Department of Pediatric Oncology/Hematology, Otto-Heubner Centre for Pediatric and Adolescent Medicine (OHC), Charité – Universitätsmedizin Berlin, corporate member of Freie Universität Berlin, Humboldt-Universität zu Berlin and Berlin Institute of Health, Berlin, Germany; 2 Unit of Integrative Pediatrics, Department of Pediatrics, Faculty of Medicine, University of São Paulo, São Paulo, Brazil

**Keywords:** pediatric oncology, pediatric complementary and integrative health, external anthroposophic therapies, musictherapy, qualitative study

## Abstract

**Background:**

Despite an increase in use of pediatric complementary and integrative health (PCIH), many healthcare professionals still have an inadequate understanding of such practices and consider their use inappropriate, which might thwart implementation processes. In a qualitative interview study we examined the feedback of conventional healthcare professionals about the integrative practices provided to pediatric patients by an integrative team in a pediatric oncological hospital.

**Methods:**

Fifteen semi-structured interviews were carried out with various conventional healthcare professionals in an university pediatric hospital in São Paulo, Brazil. The interviews were audio-recorded, transcribed and pseudonymized. DSCsoft^®^ and MAXQDA^®^ software assisted in a profound qualitative analysis using the collective subject discourse and thematic analysis method in order to display participants’ perspectives on PCIH and the project in their hospital.

**Results:**

Interviewees acknowledged their lack of knowledge about PCIH practices and reflected on the limits of their care as well as on new possibilities PCIH could offer. PCIH was perceived by interviewees as an effective supportive tool of care to promote patients’ wellbeing, assist overall compliance, strengthen cooperation between professionals, children and their relatives and hence facilitated general patient care. Since PCIH was implemented in their clinic, perceptions led interviewees to wish for increased PCIH offering and a more profound integration of its therapists into the standard of care.

**Discussion:**

The coexistence of integrative and conventional practices in the conventional healthcare setting is important to give visibility to the possibilities offered by the integrative pediatrics field. Regular and constant encounters with integrative practices, as well as information access seem crucial to reach a wider openness for PCIH and subsequently a broader application and dissemination of it.

## Introduction

Complementary and integrative health (CIH) broadens the repertoire of conventional Western allopathic healthcare by combining, integrative and complementary practices with the best of scientific evidence to expand the traditional healthcare model into a psychosocial model of healthcare emphasizing the treatment of the whole person.^1^

With the integration of CIH in the Brazilian public health care system, these practices are now part of the official Brazilian healthcare provision policy (Federal Decree No. 5813).^2^ Rising demand by patient for such practices, depending on patient type, region, clinical setting and therapy, substantiates the decision for government support.^3-5^ At the same time, a growing number of studies and evidence emphasize the benefits of CIH in various illnesses and conditions in children.^6-8^

The use of CIH in Brazil is concentrated in primary care and less than 5% occurs in the tertiary and specialized healthcare services.^9^ A study from 2019 found a 4.1% prevalence of CIH use in the Brazilian general public.^3^ Another study with 70 diabetes type 1 Brazilian pediatric patients showed that 41.5% of them used some type of CIH and most of them (69%) did not inform their doctors about this use.^4,10^ Health education inclusive of CIH is only emerging and while there are some researches in the area, only a few publications exist.^11^

Outside of Brazil, use of these practices in pediatrics is significant, as the 2012 US-American National Health Interview Survey revealed that 12% of children are treated with complementary therapies.^12^ In studies about specific chronically ill populations, up to 80% of study participants have reported complementary therapy use.^13,14^ However, less than 5% of pediatricians were reported to have enough knowledge to inform patients and families about the use and the different modalities of CIH.^15^

In 2017, the American Academy of Pediatrics recognized the increasing use of complementary and integrative practices for children and the subsequent need to provide reliable information and high-quality clinical resources to support pediatricians.^12^

Although there is growing interest and use of CIH worldwide, it is evident that there are still many obstacles to implementation: little or no funding in the public health system exists, reimbursements from health insurance companies are rare; good quality research in the field is lacking, and healthcare providers are skeptical. Recognizing these obstacles is important to improving implementation outcomes.^13^

Experiences of implementing CIH in clinical settings are frequently reported together with surveys. Most of these experiences demonstrate the prevailing lack of information among healthcare professionals about CIH. Coordination and cooperation between conventional healthcare providers and CIH professionals could improve patient care by opening communication, increasing mutual understanding, and increasing patient satisfaction and safety.^14,16^

Strong affiliations between CIH and hospitals, healthcare systems, and health schools demonstrate that integrative health has already established as part of healthcare in many developed countries, such as the United States, Canada, Switzerland, Sweden, and Germany.^17,18^ However, in other countries, including Brazil, successful integration of CIH into the education for healthcare professionals remains underreported and lacking.^19,20^ Almost half of the CIH use in Brazil occurs outside the official health care system.^4^ Lack of trained professionals, inadequate financial resources, limited knowledge on implementing and maintaining such practices, and incomplete scientific evidence on practices could explain the only partial integration of CIH into the public sector.^21^

At the Institute of Children and Adolescents at the clinics of the Faculty of Medicine, University of Sao Paulo, in partnership with the Charité - Universitätsmedizin Berlin, a Unit of Integrative Pediatrics (UPI, Portuguese acronym) was founded in 2017. Initially, a variety of integrative approaches and therapies were implemented and evaluated in the pediatric department in a pilot project phase.^22,23^ In 2021, the UPI was restructured according to findings from the pilot evaluations in order to fully implement a refined and focused pediatric complementary and integrative health (PCIH) concept for treatment of children in the pediatric department. The UPI’s work aims to promote mental health support, child-family friendly unit care, mitigation of medicines with problematic side effects, and giving patients and their parents an active part in the handling of the illness.^24^ With this concept fully implemented, UPI offers regular external anthroposophic therapies and music therapy to children with chronic and complex conditions in an inpatient setting in the Childhood Cancer Institute ward of the University of São Paulo. Additionally, a mindfulness-based program for ambulatory patients is offered throughout the pediatric clinics.^25^

In this article, we perform a qualitative analysis derived from semi-structured interviews conducted with conventional pediatric oncology healthcare providers. The objectives were as follows:

(a) to perceive their previous knowledge and experiences with the integrative field
(b) to present their perceptions and expectations about the implementation of a unit of integrative pediatrics in a pediatric hospital environment
(c) to identify barriers and facilitators for the implementation of a unit of integrative pediatrics in a pediatric hospital environment.

This study is part of a larger multi-year assessment comprising quantitative and qualitative evaluations for the design of an implementation manual to guide future PCIH hospital implementation in Brazil.

## Methods

### Study Design

This research was conducted from October to November 2021 at the end of implementation phase from May to October 2021 of the multi-year project to develop an evidence-based implementation plan for PCIH practices.

This article focuses on the implemented external anthroposophic therapies (EAT), and music therapy for inpatient children with oncological illnesses. EAT comprise external applications of essential oils in the form of embrocations, poultices, wraps, or compresses. In this program mainly rhythmic embrocations with arnica, rose, or lavender oil were used. Depending on the children’s needs and medical indications, these were applied on specific parts of the body or throughout. The program is structured as follows in short: 2 EAT trained nurses and 2 music therapists offer individualized integrative practices to the patients on the ward each twice a week. Patients were selected for treatments in consultation with the medical team on a weekly basis. This selection was carried out under the leadership of one of the authors, who is both a pediatrician and clinical coordinator on ward rounds. Patients chosen for music therapy treatment experienced communication or socialization impairments in addition to long term hospital stay. EAT were given to address physical or mental symptoms that were difficult to manage with medication only, for example, pain, fatigue, insomnia, nausea/vomiting, stress, anxiety, or depression. After each session, the healthcare provider documented their personal experience, symptom progress or regression with a symptom scale and in personal notes. Monthly meetings with the UPI’s team are used to discuss the project’s experiences, progress and difficulties. These data will be published elsewhere.

Ethical approval was granted by the Ethical Committee of the University of São Paulo (52082521.4.0000.0068).

### Sampling

Fifteen interviews were carried out with multidisciplinary oncological healthcare providers. With a convenience sampling strategy, participants were eligible if they were part of the routine healthcare staff and were willing to be interviewed. All personnel joining in on weekly multidisciplinary rounds to discuss patients’ conditions were invited to participate, ensuring they had been in contact with children receiving integrative treatments. Written consent was obtained for all participants. After interviewing all available personnel, the sample size was considered complete. No professional declined being interviewed.

### Data Collection

The interviews were conducted in person between October and November 2021 at the research site by AM, a psychologist with a master in Public Health and trained in qualitative research. A semi-structured interview guideline with 12 open questions invited participants from different backgrounds to speak freely about their perspectives. The questions were designed to answer the research topics while encouraging participants to give extensive answers with little interruption.

### Data Analysis

Interviews were audio-recorded, transcribed verbatim, and pseudonymized.

Data was analyzed using the DSC (Discourse of the Collective Subject)^26^ technique and supported by the DSCsoft^®^ computer program as a data processing and analysis software to reach the study objectives. The DSC technique aims to simultaneously analyze the interviewee’s individual narrative, as well as compare this to the collective reference: similar thoughts and concepts expressed by multiple participants. These data were subsumed in general ideas.

To guarantee an even more rigorous analysis of the data, extensive answers to questions were then coded line by line using MAXQDA^®^ software and thematic analysis approach^27^ allowing for a deeper understanding of each individual statements and subtopics. Deductive themes deriving from the original interview questions were combined with salient themes that emerged during the analysis.

The qualitative analysis was undertaken independently by 3 qualitative researchers (AM, DS, and MT) and regularly discussed to increase validity.

## Results

Fifteen interviews, varying between 30 and 90 minutes in length were conducted.

The following categories emerged from the analysis:

(1) Restructuring care: Knowledge, limits and possibilities;
(2) Perceived effects in patients, relatives and professional life; and
(3) Consolidating CIH as part of the integrative care, as well as intertwining subcategories.

### Restructuring Care: Knowledge, Limits, and Possibilities

The health professionals reported that the theoretical and practical background they had at PCIH resulted from experiences they had in the previous project or elsewhere. PCIH was defined by interviewees as a set of individual practices that have therapeutic value despite not adhering to conventional clinical practice. The individuality of patients as well as promoting quality of life, self-knowledge, and self-care were mentioned as key elements while defining the term. However, providers admitted that their professional qualifications were not applicable to the skills needed for PCIH care provision (Table 1, subcategory 1.1: *Professional expertise on PCIH*).

**Table 1.** Statements From the Health Professionals Concerning the Category “Restructuring Care: Knowledge, Limits, and Possibilities.” São Paulo, Brazil. 2022.

Category 1: Restructuring care: Knowledge, limits, and possibilities
*1.1 Professional expertise on PCIH*
General ideas:
1. “Non orthodox” treatments but with therapeutic value
2. Consider the individual as a whole
3. Promotion of comfort and life quality
4. Patients’ self perspective, to receive the treatment in a positive and potentially more healing way
Examples:
“These are complementary therapies applied at the same time as curative therapies and will provide more comfort and improve the patient’s experience during their treatment. [. . .] They take the patient as a whole into account. They do not only consider the physical and biological dimensions, but also the psychological, social, religious dimensions and integrate all of those in a person’s life”
“I already knew about (integrative pediatrics), I had already seen the team’s activity, but it was a short contact. [. . .] We don’t have that subject at college, in residence. We don’t have any contact with it, but just from these few months since the work has begun, we can see the importance [of PCIH] to the patients and how that affects their care.”
“As far as I can tell from the practice and some reading, [. . .] I may tell you that my knowledge is not enough to say much about it.”
*1.2 Limits to the professional practice within the traditional healthcare model*
Examples:
*“Here at the hospital, for children in a critical condition, it is easy to only consider the biological, technical [aspects]. We must remember that the patients and their families are not only an illness.”*
*“Integrative health and other areas, which look at the patient as a whole, highlight that we (the professionals) are not only taking care of cancer, the child is not a cancer, she/he is a human being within a family, a society, a poor heterogeneous country. So, it makes us try to broaden our horizons, I think the more people come to join us, the higher the chance we have to help and promote health.”*
*1.3 PCIH as a possibility in the transition of healthcare models*
Examples:
*“Although the traditional model here does not demand great changes, it must be complemented, and integrative medicine fills that gap. We do not want to replace traditional medicine, but to display a different way to do things, one that also works. And we’re not taking anybody’s job away, nor destroying or discrediting their profession. It’s not that, we need this complementarity.”*

Reflecting on opportunities provided by PCIH, the professionals identified the limits of their own medical practices to provide complementary care. They described their own treatment as oriented toward curing illness (instead of the patient), pointing to the limits of focusing on a clinical outcome instead of the patients’ overall wellbeing and quality of life when the child did not have a prognosis, or had a complex chronic condition. Furthermore, the professionals depicted their work as pathogenically-oriented, based on invasive routines and painful procedures instead of bringing comfort to the children (Table 1, subcategory 1.2: *Limits to the professional practice within the traditional healthcare model*).

Providers depicted how when they witnessed other professionals working with practices such as music therapy and anthroposophic external therapies, they observed the positive effects to the child and their family and thereby reflected on how these practices provided new possibilities for care (Table 1, subcategory 1.3: *PCIH as a possibility in the transition of healthcare models*).

### Perceived Effects in Patients, Relatives, and Professional Life

The perceptions of the effects on patients cover 3 main areas: the first addresses the need to control symptoms, such as pain. The second is regarding quality of life and treatment, when relaxation and comfort given to the patients parallel to invasive procedures, medications, and operations. The last area is related to the acknowledgment on the part of the provider that the child and/or adolescent may be empowered to decide what kind of treatment and care they want to receive (Table 2, subcategory 2.1: *Perceived effects on patients*).

**Table 2.** Statements From the Health Professionals Concerning the Category “Perceived Effects on Patients, Relatives, and Professional Work.” São Paulo, Brazil. 2022.

Perceived effects on patients, relatives and professional work
*2.1 Perceived effects on patients*
General ideas:
1. Coadjutants of conventional treatment, reduce stress and leave the patients more calm
2. Benefits to comfort, well-being and promotion of quality of life
3. Reduction of adverse side effects (pain, nausea)
4. Physiological benefits (walking, eating, drinking, speaking)
5. Drug reduction
Examples:
*“I think that when you have a team focusing mainly on the comfort, taking the individual as a whole into account that adds a quality of life and quality of care. [. . .] If the physical symptom is healed, but the emotional symptom is not, the child will feel unsafe, afraid, will have an anxiety*, for example, *and* that is *not quality of life.”*
“*I think the teenager is already afraid of everything going on. ‘Am I going to leave here? Am I going to make it? Will my illness be cured?.’ The teenagers are bald, underweight, wearing tubes, in chemotherapy, they can’t eat, they’re locked in a hospital, doubtful whether they’re going to live until next year. So, I think integrative practices are really important for their care and self-knowledge.”*
*2.2 Perceived effects on relatives and families*
General ideas:
1. Relatives appear more calm
2. Receiving practices themselves could be beneficial
Examples:
*“When a father or a mother sees their child in distress, crying, in pain, they feel it, the support person suffers twice as much. I think there’s no other way and this (PCIH) will be important.”*
*“They (the families) feel included in the treatment. [. . .] They realize their life is not only about the illness, they can experience joy and have pleasant experiences, even though they are dealing with an illness. [. . .] They (the professionals) make them feel human, as a patient, who must keep living.”*
*“I think the children and teenagers get affected by seeing their parents in despair. It is like a mirror. [. . .] Their anxiety is the same as their parents, their thoughts get mixed up. [. . .] So [PCIH] it takes the load off and makes the parents transform their fear, their energy [,,,] and [they] find a slightly more positive, calmer way to transmit something better to the children.”*
*2.3 Perceived effects on professional work*
General ideas:
1. Helping the professional to deal better with the patient
2. Having a positive influence on the relation with patients and families
Examples:
*“As soon as we (the team) realize there is less need for medication, fewer conflicts, a greater well-being between the family, the patient and even the team, I think that over time the teams will realize the importance of the group and will be more open.”*
*“Sometimes we forget there is a mother or a father, we forget it because we focus on the patient and their illness and, very often, they (the families) are left aside. [. . .] And then when we apply the PCIH and take care of the parents, they are able to cooperate much more with our assistance.”*
*“I see that the care affects me as well (as a professional). If the patient is more balanced, I think everyone can benefit.”*

Providers highlighted that the positive effects on children resonate with the parents, who experience the relief of seeing their child and/or adolescent at ease, transitioning from an environment of distress and worry to another of calm and satisfaction. The professionals perceived the practices involving parents as caregivers that have the capacity to humanistically address pain and reduce parent, and by extension, children irritability as a positive prospect (Table 2, subcategory 2.2: *Perceived effects on relatives and families*).

Health professionals stated that once they observed interactions of a child and/or adolescent and their families with PCIH, this impacted their own activities. They noticed that they were able to work in a more receptive and pleasant environment, that they had more contact with the patients and their families, were able to forge stronger bonds and provide more humane care. By acknowledging these effects, the professionals admitted how their conceptions about PCIH were changed; in turn demonstrating the effective supportive care instrument encouraging the interdisciplinary work of the Unit of Integrative Pediatrics (Table 2, subcategory 2.3: *Perceived effects on professional life*).

It is worth highlighting that an interconnection was found between the effects. The child and/or adolescent under care is able to influence the feelings of the family and vice-versa, and both lead to an improvement of the working environment, binding the team, and the patient in the motivation for assistance.

### Consolidating PCIH as Part of Integrative Care

The health professionals said that once they recognized PCIH as a useful tool and realized the positive effects in their practice and the integrative care of the family, they intended to absorb them into routine assistance. They nonetheless listed obstacles they faced, such as: structural barriers, financial barriers, lacking material, and qualified human resources; and refusal, either for resistance of families to start the practice and/or the refusal of the team, due to the perception that PCIH aim to shift practices and fill the space of the established team in a given sector (Table 3, subcategory 3.1: *Barriers to the insertion of PCIH as a support to traditional care*).

**Table 3.** Statements From the Health Professionals Concerning the Category “Consolidating PCIH as Part of Integrative Care.” São Paulo, Brazil. 2022.

Consolidating PCIH as part of integrative care
*3.1 Barriers to the insertion of PCIH as a support to traditional care*
General ideas:
1. Lack of knowledge of integrative practices
2. Lack of integration of professionals (impairs therapeutic planning)
3. Lack of human resources
4. Hygiene in relation to debilitated patients
5. Refusal by relatives (due to missing knowledge)
6. Lack of physical space
Examples:
*“The barriers are funding, material, number of personnel, physical space to work in, [. . .], the understanding from health professionals. [. . .] If we don’t grasp a different approach, all their work (by the professionals) goes down the drain, if we’re not together.”*
*“There’s a lot of suspicious people. “No, wait, why are you coming to my sector?”[. . .] I think there’s a lack of good will from those already working here to know something new. “But wait, I have always done it like this, why do I have to change? Why are they going to do it differently?” So, at times, it sounds like a personal thing. “Do you mean I’m not doing my job properly?.”*
*3.2 Strategies to tackle deadlocks for a successful integration*
General ideas:
1. Introduce the whole team to CIH concepts: Importance and effects
2. Increase the integration in the whole team through meetings and experiences
3. Expand the team
4. Increase the frequency and continuity of the practices
5. Active search for patients by integrative team
6. Increase the diversity of offered practices
7. Physical space for the integrative team
Examples:
*“I’m sure that to make it work, we must have the group working beside us. [. . .] There must be an immersion of the integrative pediatrics group, an ongoing process Therefore, the professionals have to be there, they must see the patient, assess their needs. [. . .] There must be a constant partnership, but it must be immersive, it can’t just work in short periods at the hospital.”*
*3.3. Successful integration—long-term repercussions and expectations*
General ideas:
1. Expand the attendance to be daily and constant, and attend to all departments
2. Expand the number of employees and modalities of practices
3. Deeper integration between teams
4. Research, teaching, learning, and verification of the results of the implementation
5. Decrease hospitalization time and the administration of medications
Examples:
*“I foresee shorter hospitalization periods, a better interaction with parents and children, a better relationship between professionals and patients’ parents. These are only benefits. [. . .] I foresee a decrease in analgesic doses, because there’s less focus on the illness.”*
*“I think this must be implemented much earlier, at the beginning of treatment if possible. [. . .] to accommodate a family from the beginning.”*

The professionals reported strategies, such as sharing knowledge across health professional specialties, designing the therapeutic plan across teams, allowing for the immersion of PCIH through communication and alignment of practices from diagnosis into daily care. It was mentioned that the implementation should be a continuous process instead of single and limited interventions (Table 3, subcategory 3.2: *Strategies to tackle deadlocks for a successful integration*).

The professionals signalled that they believe PCIH may reduce the length of hospitalization and cost of treatment or drug use, in addition to improving the integration between teams, and between the family and team. Given what they witnessed, they claimed to expect a scaling up of integrative care along with a multidisciplinary team, that would be applicable at diagnostics, for children in serious conditions at intensive care units, and/or for palliative care (Table 3, subcategory 3.3: *Successful integration—long-term repercussions and expectations*).

## Discussion

This study aimed to get a deeper understanding of the healthcare professionals’ perspective on the implementation of PCIH in a pediatric oncological center. Understanding the perception of healthcare providers on CIH may be a strategy to guide the implementation of such practices in a hospital environment.

The conducted interviews generally demonstrated an open attitude of staff toward the implemented practices. The health care professionals reported perceived improved wellbeing and compliance as the main effects in patients, leading parents to become more calm and amenable. The professionals described how these benefits had a direct impact on their own treatment and relationship with the children and their families. Similar effects on patients, families and healthcare professionals’ general well-being have been demonstrated in previous studies.^23,28^

General knowledge of PCIH practices prior to integration were insufficient, which is consistent with previous studies where less than 5% of pediatricians had adequate knowledge of CIH.^15^ Although the staff did not have much prior knowledge of CIH, they demonstrated interest in further integration and augmentation of the practices. According to the interviewees, this could be potentially achieved by expanding knowledge of integrative practices, hence showing their importance and efficacy in caring for children and their relatives.

In our study, healthcare providers witnessed the PCIH effects firsthand and described how this opened them up and to reflecting on their standard working procedures. Conventional healthcare providers show general interest in PCIH, especially in environments where they are confronted with heavier caseloads and patients’ demands.

A US-American survey found that only 24% of pediatric healthcare professionals referred their patients to CIH practitioners, even though 67% thought it could be beneficial in treating symptoms, and 59% thought it could improve patient satisfaction.^29^ Asked why, practitioners highlighted their lack of information about the practices. This finding is corroborated by other studies worldwide.^30-32^ In a Swiss survey, physicians seemed to rely more on clinical evidence and research, while nurses seemed to be convinced more by personal experience suggesting that information about practices should be tailored to expertise and experience.^33^

The existing literature indicates that formal education, didactic sessions, workshops, self-learning modules, clinical observation, and clinical practice can be adopted as implementation strategies in an urban pediatric teaching hospital.^34,35^ Improving the knowledge of conventional health care providers could be crucial to create a high willingness within the team to carry an implementation process and maintain it in the future.

Considering the psychosocial needs of children with cancer, existing guidelines describe the necessity for efficient coping strategies for illness-related burdens, such as psychosocial interventions, artistic, or supportive therapies.^36^ PCIH practices might be able to provide for these needs and could play an important role in a comprehensive patient care. From the patients’ perspective, improving the quality of life is important and an often-undermined aspect, that should be considered.^37,38^

Overall PCIH use is often increased in patients from a higher educational or income level.^39,40^ In this current project, PCIH practices are offered in an urban, public medical university context open to the general public in Brazil. Especially in the context of health promotion and psychosocial needs, UPI’s project has the potential to make a great contribution to the health of patients who could not afford these practices for economic reasons, especially in a country with great economic inequality.

At the same time, the introduction of an integrative medicine professional focused on delivering integrative care to patients working together with the regular care team may help with challenges, such as lack of CIH education, time constraints, and high workload.^41,42^

Successful implementation must be based on clinical care, education, and scientific research. It should include leaders of the hospital, such as physicians, nurses, and administrative staff, as well as professionals with an integrative health orientation. The implementation process can take some years and creating an integrative department may be necessary.^43^ The initiation of an integrative health program may require a significant initial investment and a motivated team from the outset.^44,45^ Subsequently, it is important to sustain this initial motivation to ensure a real integration of CIH practices for the maintenance and dissemination, leading with positive examples and maintain commitment in the integrative and conventional team.

### Study Limitations

The interviews were performed in a setting with a very homogeneous healthcare team specialized in pediatric oncologic patients which may not represent the reality of other pediatric centers. In addition, it was a single center study, which is why we needed to pseudonymize quotes. In this study we opted for a qualitative design as this is particularly well suited for recording perceptions and experiences. Further investigations might include quantitative data collection on patients’ symptoms, quality of life, and parents’ stress levels to measure a clinical outcome of the practices.

## Conclusion

Implementing PCIH in conventional health services may allow health professionals to realize how PCIH may support their patients’ healthcare and health promotion. Interviews with healthcare providers with little previous knowledge of CIH demonstrated openness for PCIH adoption and awareness of the potential of PCIH for patients’/families’ and the healthcare team’s well-being. Direct experience with PCIH may lead to higher motivation to implement and disseminate integrative and complementary practices in the hospital context.
